# Development of Novel Epigenetic Anti-Cancer Therapy Targeting TET Proteins

**DOI:** 10.3390/ijms242216375

**Published:** 2023-11-15

**Authors:** Hyejin Kim, Inkyung Jung, Chan Hyeong Lee, Jungeun An, Myunggon Ko

**Affiliations:** 1 Department of Biological Sciences, Ulsan National Institute of Science and Technology, Ulsan 44919, Republic of Korea; bpk0191@unist.ac.kr (H.K.); ikjung610@unist.ac.kr (I.J.); 2 Department of Life Sciences, Jeonbuk National University, Jeonju 54896, Republic of Korea; chlee9243@jbnu.ac.kr; 3 Center for Genomic Integrity, Institute for Basic Science, Ulsan 44919, Republic of Korea

**Keywords:** epigenetic dysregulation, TET proteins, DNA methylation, hydroxymethylation, cell-based screening, mitoxantrone, cancer therapy

## Abstract

Epigenetic dysregulation, particularly alterations in DNA methylation and hydroxymethylation, plays a pivotal role in cancer initiation and progression. Ten-eleven translocation (TET) proteins catalyze the successive oxidation of 5-methylcytosine (5mC) to 5-hydroxymethylcytosine (5hmC) and further oxidized methylcytosines in DNA, thereby serving as central modulators of DNA methylation–demethylation dynamics. TET loss of function is causally related to neoplastic transformation across various cell types while its genetic or pharmacological activation exhibits anti-cancer effects, making TET proteins promising targets for epigenetic cancer therapy. Here, we developed a robust cell-based screening system to identify novel TET activators and evaluated their potential as anti-cancer agents. Using a carefully curated library of 4533 compounds provided by the National Cancer Institute, Bethesda, MD, USA, we identified mitoxantrone as a potent TET agonist. Through rigorous validation employing various assays, including immunohistochemistry and dot blot studies, we demonstrated that mitoxantrone significantly elevated 5hmC levels. Notably, this elevation manifested only in wild-type (WT) but not TET-deficient mouse embryonic fibroblasts, primary bone marrow-derived macrophages, and leukemia cell lines. Furthermore, mitoxantrone-induced cell death in leukemia cell lines occurred in a TET-dependent manner, indicating the critical role of TET proteins in mediating its anti-cancer effects. Our findings highlight mitoxantrone’s potential to induce tumor cell death via a novel mechanism involving the restoration of TET activity, paving the way for targeted epigenetic therapies in cancer treatment.

## 1. Introduction

Epigenetics, a regulatory mechanism altering gene expression without changing nucleotide sequences, is critical for diverse biological processes, including gene transcription, chromatin organization, and X-chromosome inactivation [1]. DNA methylation, a well-explored epigenetic modification catalyzed by DNA methyltransferases, maintains genomic stability; its imbalance leads to genomic instability and cancer [2]. The ten-eleven translocation (TET) proteins, comprising TET1, 2, and 3, are Fe(II)- and 2-oxoglutarate (2-OG)-dependent dioxygenases that finetune DNA’s methylation status by oxidizing 5-methylcytosine (5mC) to 5-hydroxymethylcytosine (5hmC), 5-formylcytosine (5fC), and 5-carboxylcytosine (5caC) [3,4,5]. TET-catalyzed oxidized methylcytosines inhibit the function of maintenance DNA methyltransferase, enabling the progressive dilution of 5mC following DNA replication, a process known as “passive DNA demethylation” [5,6,7]. Furthermore, TET proteins are also essential for active DNA demethylation: 5fC and 5caC are recognized by thymine DNA glycosylase (TDG)/base excision repair (BER) machinery to be converted to unmethylated cytosines [5,6,7]. TET-assisted DNA demethylation exerts profound effects on gene expression by impacting the local chromatin structure and transcription factor accessibility at enhancers and promoters, thereby securing proper cell lineage differentiation, survival, and proliferation [5,6,7].

Dysregulation of TET proteins contributes to tumor growth and metastasis, observed in various types of cancers. TET loss of function (LOF) via genetic mutations or epigenetic dysregulation is prevalent in a wide range of hematological malignancies and solid tumors [5,8,9]. In particular, TET2 LOF mutations are recurrent in patients with myeloid and lymphoid malignancies, and mice with the targeted inactivation of their TET proteins in hematopoietic cells succumbed to diverse types of hematological malignancies [3,8,10,11]. The murine models generated so far and their phenotypes are summarized in Ref. [5]. All three members of the TET family are mutated in colon cancer, and *TET2* mutations and deletion are also observed in clear cell renal cell carcinoma and ovarian carcinoma patients [12,13,14]. However, TET LOF is generally achieved without genetic alterations in many solid tumors [9,15,16,17,18,19].

Notably, the restoration of TET enzyme expression or function inhibits cancer progression [18,20,21,22,23,24,25,26,27]. The overexpression of TET1 inhibited colon cancer formation in mice by derepressing inhibitors of the WNT pathway such as DKK3 and DKK4 [22]. Restoration of TET2 reversed abnormal hematopoietic stem and progenitor cell (HSPC) self-renewal and inhibited myeloid leukemia development in mice [25]. Furthermore, in metastatic cancers such as breast and ovarian cancer, low TET2 and 5hmC levels were positively correlated with the resistance of cancer cells to chemotherapeutic drugs such as PARP inhibitor olaparib and alkylating agent cisplatin [28]. However, elevation in 5hmC levels enhanced the recruitment of APE1, a base excision repair-associated apurinic/apyrimidinic endonuclease, to the stalled replication fork, thereby promoting the degradation of the stalled replication fork and limiting cancer cell growth [28]. In hepatocellular carcinomas, reduction in TET2 and thereby 5hmC induced chemotherapeutic resistance through the inhibition of histone acetyltransferase P300/CBP-associated factor (PCAF) and hyperactivation of the AKT pathway [29].

Several pharmacological activators exhibit direct or indirect TET agonism, demonstrating clinical efficacy in cancer treatment. Vitamin C accelerates the recycling of Fe(II), an essential cofactor of TET enzymes, thereby augmenting TET activity [30,31]. Consistent with frequent TET inactivation in cancers, physiological vitamin C levels are markedly reduced in many cancer patients, compared with healthy individuals [32,33,34]. Many studies have reported that vitamin C increases 5hmC in cancer cell lines including leukemia, hepatocellular carcinoma, and colorectal cancer [35,36,37,38,39], demonstrating therapeutic efficacy, particularly in the treatment of hematological cancers [25,27]. Additionally, FDA-approved drugs like ivosidenib [40] and enasidenib [41] targeting IDH1 and IDH2, respectively, demonstrated clinical efficacy in IDH-mutated AML [42,43,44], glioma [45,46,47], and cholangiocarcinoma [48,49], either alone or in combination. Although it is not yet clear whether these IDH inhibitors antagonize tumor growth by targeting TET proteins, they function as indirect TET agonists by reducing levels of the oncometabolite 2-hydroxyglutarate (2HG), a competitive inhibitor of TET enzymes [42]. As vitamin C and IDH inhibitors influence the activity of a broad range of α-KG-dependent enzymes, including TET proteins, further screening efforts are required to develop more effective and specific TET agonists that are applicable to cancer therapy.

As the catalytic activity of the TET enzyme plays a key role in suppressing oncogenesis, identifying agents restoring TET activity would offer a promising avenue for novel epigenetic anti-cancer therapies. In this study, we developed a cell-based assay to identify novel TET agonists. Utilizing an inducible cell clone expressing the human TET1 catalytic domain at low levels, we conducted a primary screening, leading to the identification of mitoxantrone as a potent TET agonist. Mitoxantrone significantly increased 5hmC levels and induced cell death in leukemia cell lines in a TET-dependent manner.

## 2. Results

### 2.1. Development of an Optimal Cell Line for TET Agonist Screening

To establish an efficient cell-based screening system for TET agonists, we developed an HEK293 cell line expressing the carboxy-terminal catalytic domain (CD) of TET1 in a stable and inducible manner utilizing a tetracycline (Tet)-regulated gene expression system. While the functional significance of the amino-terminal non-CD region of the TET1 has yet to be identified, it is well established that the isolated CD of the TET1 protein possesses full enzymatic activity [50,51]. Moreover, the core CDs of TET1, TET2, and TET3 are highly conserved [52]. Notably, *TET1* is preserved in its entirety in most cancers without undergoing mutations, unlike *TET2*, which frequently undergoes somatic mutations [5,8,53]. Consequently, we opted to establish a cell line expressing TET1-CD rather than the full-length protein.

**Figure 1:**
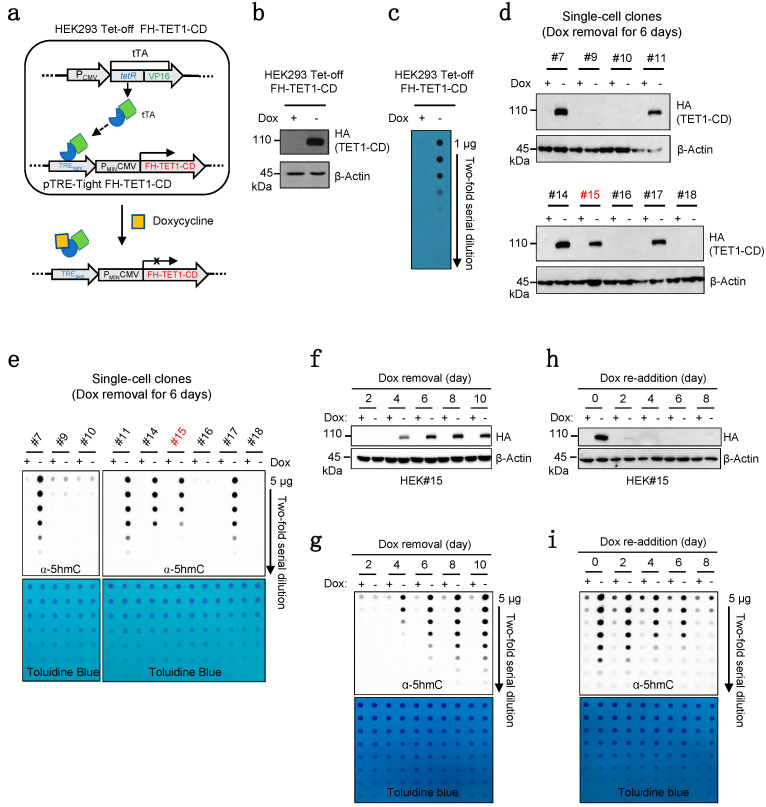
Establishment of the TET agonist screening system. (**a**) Inducible expression of the catalytic domain (CD) of TET1 was achieved by stable transfection of FLAG-HA (FH) tagged as TET1-CD in the pTRE-Tight vector into HEK293 Tet-Off cells. FH-TET1-CD expression was triggered upon the withdrawal of doxycycline (Dox) from the culture medium. (**b**) Immunoblot analysis revealed FH-TET1-CD protein levels in FH-TET1-CD-HEK293 Tet-Off cells cultured for 6 days with or without Dox, with β-actin serving as a loading control. (**c**) Determination of 5hmC levels in cells shown in (**b**). (**d**) Levels of TET1-CD in whole-cell lysates from single-cell clones derived from FH-TET1-CD-HEK293 Tet-Off cells cultured for 6 days with or without Dox. β-Actin was used as a loading control. (**e**) Determination of 5hmC levels in cells described in (**d**). Toluidine blue staining was used to monitor equivalent DNA loading. (**f**,**g**) Levels of FH-TET1-CD proteins (**f**) and 5hmC (**g**) in HEK#15 cells over specific time periods with (+) or without (−) Dox. β-Actin served as a control. (**h**,**i**) HEK#15 cells were cultured with (+) or without (−) Dox, followed by re-addition of Dox to both cultures for the indicated time points. Levels of FH-TET1-CD proteins (**h**) and 5hmC (**i**) were assessed by immunoblotting and dot blot assay, respectively. β-Actin served as a control and toluidine blue staining was used to monitor equivalent DNA loading.

We cloned the FLAG-HA (FH) tandem, tagged as TET1-CD (termed FH-TET1-CD), into a pTRE-Tight vector which induces gene expression under the control of a tetracycline-responsive promoter. This construct was stably transfected into an HEK293 Tet-Off Advanced cell line, which expresses the tTA transcription factor (Figure 1a). Upon the removal of doxycycline (Dox), a tetracycline derivative, expression of FH-TET1-CD proteins and the resultant elevation in 5hmC levels were verified through immunoblotting and 5hmC dot blot analyses (Figure 1b,c).

To minimize potential variations due to the heterogeneous cell populations with different levels of FH-TET1-CD expression, we subsequently performed single-cell cloning to select clones expressing optimal levels of FH-TET1-CD. When we assessed levels of FH-TET1-CD and 5hmC after culturing each single-cell clone in the absence of Dox for 6 days, the #15 clone (hereafter referred to as HEK15) displayed lower levels (Figure 1d,e), aligning perfectly with the requirements for TET agonist screening. Additionally, we rigorously tested the reversibility of our system in the presence or absence of Dox. Upon removal of Dox, both FH-TET1-CD and 5hmC gradually increased and stabilized for a minimum of 10 days (Figure 1f,g). In contrast, upon Dox reintroduction, FH-TET1-CD expression was immediately inhibited, concomitantly reducing 5hmC levels to their basal levels for up to 8 days (Figure 1h,i). Based on these observations, HEK15 clones after Dox removal for at least 10 days were utilized in subsequent experiments.

### 2.2. Optimization of the Screening System for TET Agonist Identification

In our pursuit of an efficient screening system for TET agonists (Supplementary Figure S2a), we quantified 5hmC levels in cells by incorporating an in-cell Western assay, a powerful quantitative technique that enables the analysis of target expression in situ using antibodies conjugated with near-infrared fluorescent dyes [52]. Prior to the screening of drug libraries, we performed antibody titration to identify staining conditions yielding optimal signals with minimal background noise (Supplementary Figure S1a). Additionally, we determined the optimal concentration of maleimide, ensuring the appropriate number of cells per well while monitoring compound-induced cytotoxicity (Supplementary Figure S1b).

**Figure 2:**
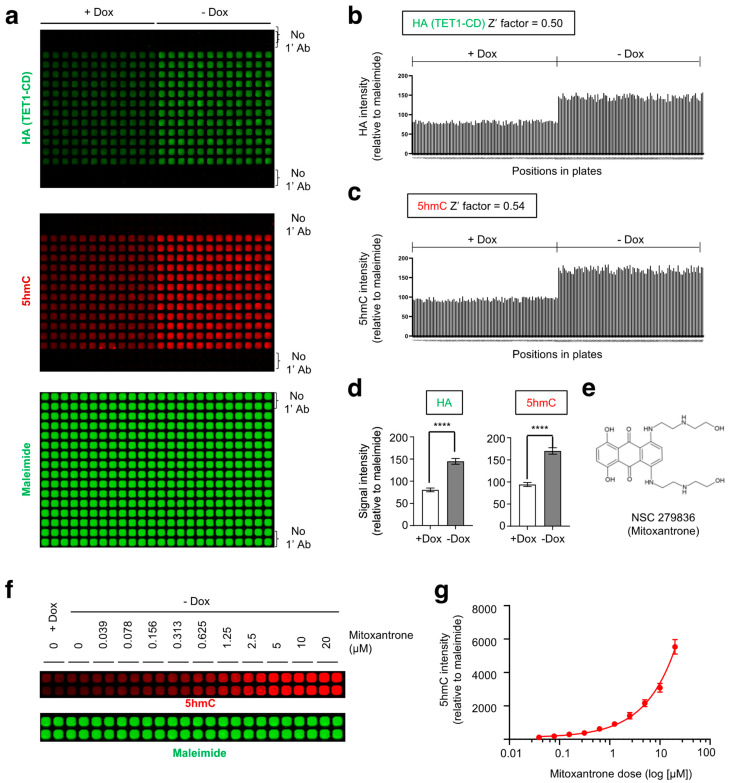
Identification of mitoxantrone as a potential TET agonist through primary screening. (**a**) HEK#15 cells (4 × 10^4^ cells per well in 50 μL) cultured with (+) or without (−) Dox were seeded and maintained in a 384-well plate. After 2 days, the plate was co-stained with antibodies specific to HA (green) and 5hmC (red). Maleimide staining was used to monitor compound-induced cytotoxicity. (**b**,**c**) Levels of FH-TET1-CD protein and 5hmC in individual wells of the 384-well plate described in (**a**). Uniform staining intensities for TET1-CD and 5hmC within individual wells were observed. The Z’ factor values are also provided. Signal intensities were normalized to the mean of the maleimide staining intensities. (**d**) Summary of the results presented in (**b**,**c**). The mean ± s.d. is shown. (**e**) Molecular structure of NSC 279836, also known as mitoxantrone. (**f**) Dose-dependent elevation in 5hmC intensities in HEK15 cells upon mitoxantrone treatment. Maleimide staining was used to monitor compound-induced cytotoxicity. (**g**) Summary of the 5hmC signal intensities shown in (**f**). The 5hmC signal intensity was calculated by subtracting the mean of the vehicle-treated group from that of the drug-treated groups, which was then normalized to the mean of maleimide staining intensities. All data are presented as the mean ± s.d. The *p*-values were determined by unpaired Student’s *t*-test. **** *p* ≤ 0.0001.

To further ensure the functionality of our established screening system, cells were cultured in a 384-well plate for two days. Building upon our previous experiments (Figure 1), we utilized HEK15 cells deprived of Dox for at least 10 days ((−) Dox). As a negative control, cells treated with Dox for a minimum of 8 days ((+) Dox) were also included. As expected, FH-TET1-CD expression and elevated 5hmC levels were observed exclusively in (−) Dox cells (Figure 2a). Notably, clones with inducible FH-TET1-CD expression displayed uniform 5hmC staining intensities within individual wells, although those lacking FH-TET1-CD expression exhibited uniform background signals (Figure 2b–d), making the system ideal for high-throughput screening. Given the uniform cell population addressed by this assay, summing the total 5hmC signal from each well was deemed acceptable. These signal intensities were individually corrected for background noise.

To validate the robustness of our screening assay, we calculated the Z’ factor value, a measure of variability between two datasets [54]. The Z’ factor was derived from the formula [55]:

$$Z’=1-\frac{3\left(\sigma_{c+}+\sigma_{c-}\right)}{\left|\mu_{c+}+\mu_{c-}\right|\;}$$

where σ_c+_ represents the standard deviation of positive controls (Dox-removed HEK15 cells), σ_c−_ represents the standard deviation of negative controls (Dox-treated HEK15 cells), μ_c+_ represents the mean of positive controls, and μ_c−_ represents the mean of negative controls. A Z’ factor of 0.5 or higher signifies a suitable difference between the signal and background with minimal data variability [56]. Through rigorous evaluation, our selected staining condition demonstrated a Z’ factor of 0.5 for HA staining and 0.54 for 5hmC staining (Figure 2b,c), confirming the robustness of our assay. Together, these results underscore the feasibility of our imaging system to quantify TET1 catalytic activity, establishing it as a robust method for identifying TET modulators by measuring 5hmC levels.

### 2.3. Identification of Potential TET Agonists through Primary Screening

To perform primary screening for novel pharmacological TET agonists, we obtained a library of 4533 compounds from the Developmental Therapeutics Program (DTP) of the National Cancer Institute (NCI), Bethesda, USA. Our selection criteria focused on maximizing diversity throughout the collection and ensuring favorable physicochemical properties, including solubility, reduced toxicity, and heightened stability—criteria previously validated by the DTP.

The library used for our screening comprised distinct sets (Supplementary Figure S2b): the Mechanistic Set (derived from 37,836 open compounds and containing only those exhibiting broad growth inhibitory effects on the NCI’s human tumor 60 cell line screening); the Approved Oncology Drugs Set VIII (encompassing the latest FDA-approved anti-cancer drugs for cancer research); Diversity Sets IV/VI (derived from approximately 140,000 compounds and containing compounds meticulously chosen through Chem-X (Oxford Molecular Group, Oxford, UK) and Catalyst (Accelrys, San Diego, Inc., San Diego, CA, USA) programs to ensure pharmacologically desirable features for structure-based hypothesis generation); and Natural Products Set IV (comprising compounds selected from the DTP Open Repository selection of 140,000 compounds based on their origin, purity, structural diversity, and availability).

To evaluate the impact of these compounds on TET activity, HEK15 cells deprived of Dox were treated with each compound in 384-well plates in duplicate (Supplementary Figure S2c). Subsequently, cells were immunostained for 5hmC and HA after two days, providing quantitative data reflecting changes in their levels relative to vehicle treatment. TET agonist candidates were chosen, focusing on compounds with a standard deviation (s.d.) greater than 4 in comparison to the mean effect induced by the compounds in each plate. Through this stringent selection process, NSC279836 (mitoxantrone) and NSC36758 (toluidine blue O, TBO) emerged as the most potent TET agonists (Figure 2e and Figure S2d,e). Consistently, both compounds significantly potentiated 5hmC induction in HEK15 cells upon removal of Dox, as assessed by a dot blot assay (Supplementary Figure S2f).

Mitoxantrone is a type II topoisomerase inhibitor known to interfere with DNA synthesis and repair by being inserted between DNA bases [57]. To further address the relationship between 5hmC responsiveness and mitoxantrone exposure, we conducted a dose–response analysis. Upon treatment with mitoxantrone, 5hmC intensities in HEK15 cells increased in a dose–response manner, corroborating its agonistic effect on the activity of TET1 enzymes (Figure 2f,g). Likewise, TBO also displayed a similar dose-dependent effect in HEK15 cells (Supplementary Figure S2g–i).

### 2.4. Validation of the Identified Compounds as TET Agonists

**Figure 3:**
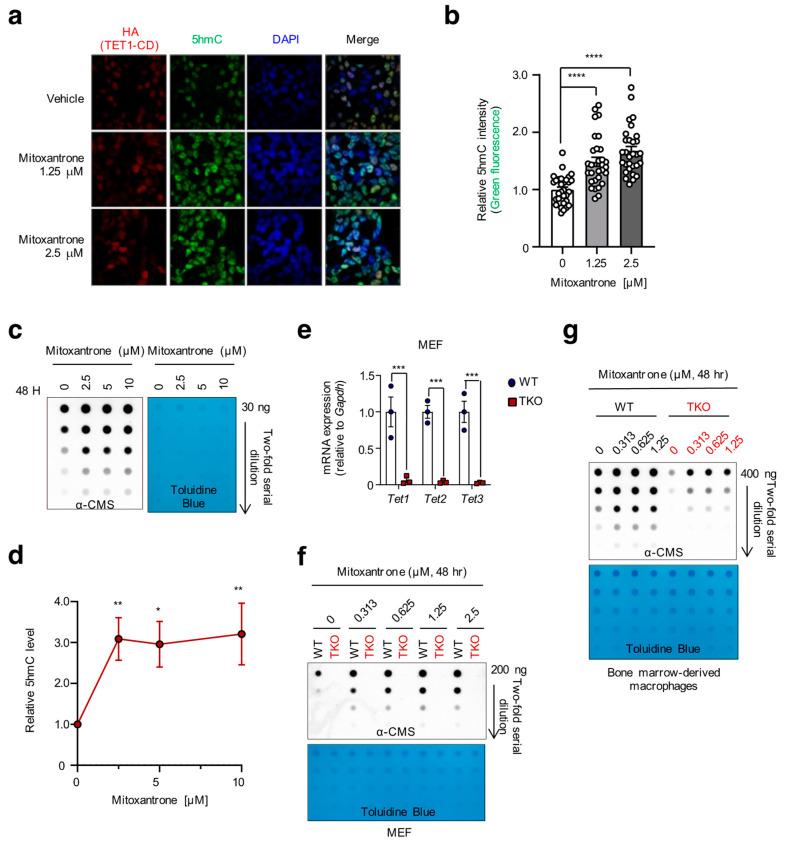
Mitoxantrone augments 5hmC levels in a TET-dependent manner. (**a**) Immunocytochemistry revealed the mitoxantrone-induced increase in 5hmC levels. HEK#15 cells, cultured without Dox, were treated with mitoxantrone at the indicated concentration and co-stained with antibodies specific for the HA epitope (red) and 5hmC (green). DAPI (blue) indicated nuclear staining. (**b**) Quantification of 5hmC intensities shown in (**a**). (**c**) Genomic DNA purified from HEK#15 cells treated with mitoxantrone as indicated was treated with bisulfite to convert 5hmC to cytosine 5-methylenesulfonate (CMS). CMS was quantified by dot blot assay using an anti-CMS antibody. Toluidine blue staining was used to monitor equivalent DNA loading. (**d**) Summary of the 5hmC intensities shown in (**c**). (**e**) Quantitative RT-PCR was performed to assess the levels of *Tet1*, *Tet2*, and *Tet3* mRNAs relative to *Gapdh* in WT and TET triple knockout (TKO) MEFs (*n* = 3). (**f**,**g**) WT and TET TKO MEFs (**f**) or BMMs (**f**) were treated with increasing concentrations of mitoxantrone for 48 h, followed by quantification of 5hmC by anti-CMS dot blot. Toluidine blue staining was used to monitor equivalent DNA loading. All data are presented as the mean ± s.d. The *p*-values were determined by unpaired Student’s *t*-test. * *p* ≤ 0.05, ** *p* ≤ 0.01, *** *p* ≤ 0.001, and **** *p* ≤ 0.0001.

To further validate the selected compounds, we employed multiple assays to confirm their effects. First, immunocytochemistry clearly demonstrated heightened 5hmC levels within the nuclei of mitoxantrone-treated cells compared to their vehicle-treated counterparts (Figure 3a,b). Moreover, a dot blot assay in HEK15 cells showed that mitoxantrone significantly elevated 5hmC levels in genomic DNA in a dose-dependent manner, further confirming its impact (Figure 3c,d).

To address the specificity of mitoxantrone’s activity, we generated mouse embryonic fibroblast (MEF) and primary bone marrow-derived macrophage (BMM) cells derived from *Tet1^fl/fl^ Tet2^fl/fl^ Tet3^fl/fl^ ERT2-Cre* mice [58]. To induce simultaneous deletion of all three *Tet* genes, MEFs were transduced with retroviruses expressing Cre recombinase (Figure 3e). BMMs were treated with 4-hydroxytamoxifen, which binds to ERT2-Cre in the cytoplasm to induce its nuclear translocation and deletion of floxed alleles as described previously [58]. Then, we conducted dose–response experiments in wild-type (WT) and TET triple knockout (TKO) cells. As anticipated, mitoxantrone robustly induced 5hmC levels in WT MEFs and primary mouse BMM cells whereas it exhibited no effects in TKO cells (Figure 3f,g), underscoring its dependency on functional TET proteins to increase genomic 5hmC levels. Similarly, TBO also displayed a dose-dependent increase in genomic 5hmC in a TET-dependent manner, as assessed by immunocytochemistry and dot blot analyses (Supplementary Figure S3). These results indicate the effectiveness of our newly devised screening system, establishing its utility for the efficient identification of a potent TET agonist.

### 2.5. Mitoxantrone Induces Cancer Cell Death via TET Activation

Mitoxantrone, a prominent member of the synthetic anthracenedione class, emerged as a breakthrough due to its diminished cardiotoxicity compared to doxorubicin, a well-known anti-cancer drug. Initially FDA-approved for adult acute myeloid leukemia in 1987 [59], its applications expanded to hormone-refractory prostate cancer in 1996 [60] and multiple sclerosis in 2000 [61]. This compound is a DNA-reactive agent that intercalates into DNA and exerts its primary action by inhibiting topoisomerase II, disrupting both DNA replication and RNA synthesis, leading to cancer cell demise [62].

**Figure 4:**
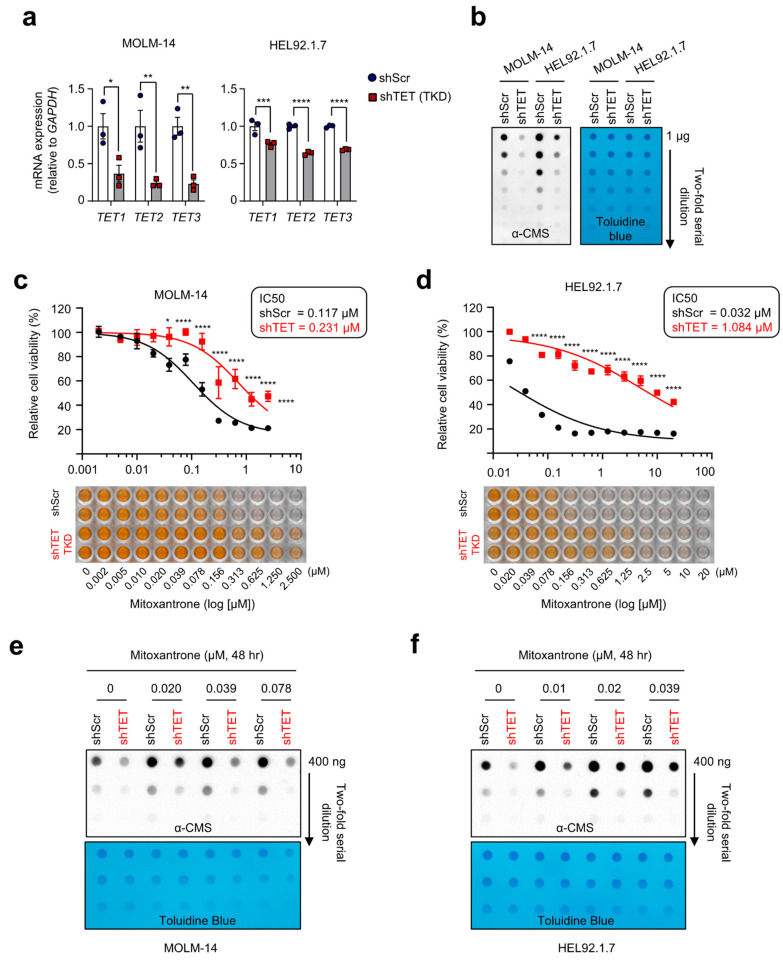
Mitoxantrone induces cancer cell death via TET activation. (**a**) Quantitative RT-PCR was performed to assess *TET1*, *TET2*, and *TET3* mRNA levels relative to *GAPDH* in control and TET triple knockdown (TKD) MOLM-14 and HEL92.1.7 cells (n = 3). (**b**) Quantification of 5hmC by anti-CMS dot blot in cells shown in (**a**). Toluidine blue staining was used to monitor equivalent DNA loading. (**c**,**d**) The impact of mitoxantrone on leukemic cell viability. Control and TET TKD MOLM-14 (**c**) and HEL92.1.7 (**d**) leukemia cells were treated with mitoxantrone at the indicated concentrations. After 48 h, cell viability was measured using the WST-8 assay. (**e**,**f**) Dose-dependent increase of 5hmC upon mitoxantrone treatment. Control and TET TKD MOLM-14 (**e**) and HEL92.1.7 (**f**) cells were treated with mitoxantrone at the indicated concentrations for 48 h, followed by 5hmC quantification by anti-CMS dot blot. Toluidine blue staining was used to monitor equivalent DNA loading. All data are presented as the mean ± s.d. The *p*-values were determined by unpaired Student’s *t*-test. * *p* ≤ 0.05, ** *p* ≤ 0.01, *** *p* ≤ 0.001, and **** *p* ≤ 0.0001.

To find out whether mitoxantrone is capable of triggering cancer cell death in a TET-dependent manner, we generated four human leukemia cell lines (MOLM-14, KG-1, HEL92.1.7, and U937) with TET triple knockdown (TKD) by transducing them with lentiviruses expressing shRNAs against TET1, TET2, and TET3. These leukemic cell lines contained different levels of 5hmC in their genome (Supplementary Figure S4a). We confirmed the simultaneous reduction in all three *TET* mRNAs in TET TKD cell lines by quantitative PCR (Figure 4a and Figure S4b). In line with this, dot blot analyses showed that levels of genomic 5hmC were significantly diminished in the TET TKD cells compared with the controls (Figure 4b and Figure S4c). Then, these cells were treated with varying concentrations of mitoxantrone for 48 h to assess its effect on cell viability (Figure 4c,d and Figure S4d). Remarkably, mitoxantrone treatment significantly impaired cell viability, particularly in MOLM-14 and HEL92.1.7 (Figure 4c,d), both exhibiting relatively higher basal 5hmC levels compared with the other cell lines (Supplementary Figure S4a). However, this cytotoxic effect was significantly attenuated in the TET TKD cells, indicating the TET dependency for inducing cytotoxicity (Figure 4c,d). Furthermore, no cytotoxic effects were observed in U937 and KG-1 cells that contained relatively lower levels of 5hmC (Supplementary Figure S4a,d).

To find out if mitoxantrone-induced cytotoxicity is associated with TET-mediated 5hmC induction, we examined 5hmC levels following mitoxantrone treatment in a dose-dependent manner. As anticipated, mitoxantrone escalated genomic 5hmC levels in the MOLM-14 and HEL92.1.7 cell lines, more effectively in control cells compared with TET TKD cells (Figure 4e,f), suggesting that mitoxantrone may induce cell death by stimulating TET enzymatic activity. Similarly, TBO also potentiated 5hmC production and cytotoxicity in MOLM-14 and HEL92.1.7 cells in a TET-dependent manner (Supplementary Figure S5). Together, these findings strongly suggest that mitoxantrone and TBO antagonize cancer cell viability by augmenting 5hmC levels in a TET-dependent manner, pointing to its potential as a promising epigenetic therapeutic agent in cancer treatment.

## 3. Discussion

Given the previous research highlighting the potential of TET modulation in antagonizing cancer cell growth, the prospect of TET modulators as anti-cancer drug targets has become increasingly promising. Despite this potential, the identification of TET enzyme regulators has remained limited, underscoring the need for more innovative screening methodologies. Here, we established an immunohistochemistry-based screening system designed for the high-throughput identification of novel TET agonists, offering a direct assessment of 5hmC levels within cells. Our cell-based screening system offers a distinctive advantage by enabling immediate confirmation of the compound’s activity on target molecules in addition to its cellular entry and cytotoxicity. To this aim, we created a stable cell line expressing the TET1 catalytic domain at low levels, permitting quantitative evaluation of TET enzymatic activity via in-cell Western analysis.

In this study, our screening approach successfully identified mitoxantrone as the most potent TET agonist from a library of 4533 compounds curated by the NCI’s DTP. Mitoxantrone significantly elevated 5hmC levels in HEK293 Tet-Off FH-TET1-CD cells, a finding corroborated by secondary assays including dot blot and immunohistochemical analyses. Through dose–response experiments with TET-deficient mouse MEFs and primary BMMs, we validated mitoxantrone’s boosting effects on the levels of genomic 5hmC in a TET-dependent manner. As a result of a significant enhancement in 5hmC levels, mitoxantrone induced tumor cell death in leukemia cell lines, particularly those harboring higher levels of basal TET activity. These findings not only advance our understanding of TET modulation but also pave the way for novel and effective epigenetic anti-cancer therapies.

Mitoxantrone is a synthetic anthracenedione analog of doxorubicin designed to minimize cardiotoxicity [62] and operates through a unique mechanism. Mitoxantrone primarily functions by inhibiting topoisomerase II, which typically causes double-stranded DNA breaks. However, in the presence of formaldehyde, which is abundant in tumors or inflammatory sites, mitoxantrone is activated and intercalates into DNA, forming mitoxantrone–DNA adducts at CpG or CpA sites [63,64]. These adducts are known to be more cytotoxic than DNA damage associated with topoisomerase II blockade. The mitoxantrone–DNA adducts inhibit the progression of replication enzymes, thereby impeding cell division and proliferation [57]. They also block the progression of RNA polymerase during transcription, particularly at methylated sites, releasing truncated RNA transcripts and inducing inflammation [63,65]. Due to these properties, mitoxantrone is widely used as an anti-cancer therapy against breast cancer, leukemia, lymphoma, and prostate cancer [66,67,68]. Notably, mitoxantrone–DNA adduct formation is facilitated by cytosine methylation at CpG dinucleotides, and its pharmacological activity is specific to DNA methylation status [65,69]. In HCT116 cells, the cytotoxicity of mitoxantrone was significantly attenuated by approximately two-fold when DNA methylation levels were diminished by DNMT1 and DNMT3a double knockout [70]. Furthermore, mitoxantrone promoted demethylation and re-expression of cyclin D2 and 14-3-3δ, consistent with the demethylase activity of TET proteins [70]. These findings indicate that mitoxantrone possesses additional epigenetic functions that may involve modulation of TET enzymes.

A previous study revealed that idarubicin, another anthracycline-class drug featuring an amino sugar structure, enhances the chromatin accessibility of TET2 through histone eviction, leading to elevated 5hmC levels [71]. Remarkably, this induction of TET activity operates independently of its role as a topoisomerase II inhibitor. Whether mitoxantrone augments TET function by influencing chromatin architecture and cellular epigenetic status, thereby controlling the accessibility of TET enzymes, remains to be determined. Notably, despite belonging to the anthracycline drug class, mitoxantrone lacks an amino sugar structure. Thus, it is also possible that it induces 5hmC production through a unique mode of action. Further studies are warranted to elucidate the exact mechanism by which mitoxantrone modulates TET activity.

It is noteworthy that mitoxantrone exerts its therapeutic effects by augmenting 5hmC levels. To substantiate mitoxantrone’s potential as a TET agonist, however, further studies are required. For instance, whether mitoxantrone exerts different effects on distinct TET family members remains unclear. More importantly, it remains to be clarified whether mitoxantrone controls TET activity directly or indirectly. Molecular modeling techniques, akin to the study of C35 [72], would be conducive to addressing these questions and synthesizing optimized derivatives. In vivo studies using xenograft mouse models with various cancer cell lines should also be conducted to confirm its anti-cancer efficacy. Future studies will resolve these issues.

Our screening also identified TBO as another compelling TET agonist candidate (Supplementary Figures S3 and S5). TBO is a phenothiazine dye commonly utilized in histological staining and has multifaceted applications in analyzing neuronal structure, hippocampal neuronal damage, and chondrocyte morphology [73]. In clinical contexts, TBO serves as an adjunctive tool that aids in the differentiation of lesions for early diagnoses of oral and oropharyngeal cancers [74,75]. Notably, it was recently shown that TBO acts as a small molecule capable of inhibiting the proliferation of Hs 766T pancreatic cancer cells by targeting β-secretase 1 (BACE1) [76]. Pancreatic cancer, currently lacking definitive treatment options, exhibits elevated levels of APLP2 and its C-terminal fragments (CTFs) which are generated by BACE1. Intriguingly, TBO reduces APLP2 expression and BACE activity, resulting in a decrease in APLP2 CTFs in Hs 766T cells. Given the widespread overexpression of APLP2 observed not only in pancreatic cancer but also in colon, breast, prostate, and lung cancers [77,78], TBO could exhibit a global anti-tumor effect through the inhibition of APLP2/CTFs. Our study has shed light on a novel facet of TBO’s functionality: its role as a TET activator (Supplementary Figures S3 and S5). Further studies will unravel the intricate relationship between TBO’s established function as an APLP2/BACE inhibitor as well as its newly proposed role as a TET activator.

## 4. Materials and Methods

### 4.1. Materials and Small Molecule Libraries

The small molecule library utilized in this study was generously provided by the NCI/NIH DTP Open Chemical Repository. To screen for TET agonists, a combination of synthetic compounds or natural products was employed, including the Mechanistic Set (plates 4849–4857), Approved Oncology Drugs Set VIII (plates 4858–4859), Diversity Set VI (plates 4860–4879), Diversity Set IV (plates 4770–4789), and Natural Products Set IV (plates 13160330–13160331). All chemical reagents were diluted in a solution of 10% dimethyl sulfoxide (DMSO, D8418; Sigma-Aldrich, St. Louis, MO, USA) and 90% sterile DPBS without calcium and magnesium (21600-044; Gibco, Waltham, MA, USA) to achieve a stock concentration of 100 μM and stored at −20 °C. For the high-throughput screening, 5 μL of the 100 μM stock of compounds was dispensed into the individual wells of a 384-well plate, and these were then combined with 45 μL of complete media containing cells. Mitoxantrone 2HCl (CAS# 70476-82-3) and toluidine blue O (CAS# 92-31-9) were purchased from Selleckchem (S2485) and TCI America (T0571), respectively.

### 4.2. Mice

*Tet1^fl/fl^ Tet2^fl/fl^ Tet3^fl/fl^ Cre-ERT2* mice were previously described [58] and a generous gift from Dr. Anjana Rao of La Jolla Institute, USA. *Cre-ERT2* (#8085) mice were purchased from Jackson Laboratories. Mice were maintained in a temperature-controlled (22 ± 2 °C) facility under a 12 h light/dark cycle. Mice were bred and maintained in the In Vivo Research Center (IVRC) at the Ulsan National Institute of Science and Technology (UNIST, Ulsan, Korea) under specific pathogen-free conditions. All experimental protocols were approved by the Institutional Animal Care and Use Committee of UNIST (UNISTACUC-17–35, 20–23).

### 4.3. Generation of WT and Tet Triple Knockout MEFs and BMMs

For the derivation of MEFs, embryos from the *Tet1^fl/fl^ Tet2^fl/fl^ Tet3^fl/fl^ Cre-ERT2* mice were isolated at E13.5. After the heads, tails, limbs, and most of the internal organs were removed, the embryos were minced and trypsinized for 20 min and then seeded into T-75 cell culture dishes in 15 mL of complete MEF media (DMEM supplemented with 10% FBS (Hyclone, Logan, UT, USA), 1X penicillin/streptomycin, non-essential amino acids, GlutaMAX^TM^, gentamycin, and β-mercaptoethanol). Once the cells reached confluency, they were frozen or expanded for further studies. To induce simultaneous *Tet* deletion, empty pMSCV-Puro or pMSCV-Cre-Puro retroviral vectors were transfected into PlatE packaging cells, and viral supernatants were collected after 48 h and filtered through 0.45 μm low-protein binding filters. Then, MEFs were transduced with retroviruses in the presence of 8 μg/mL polybrene and selected with 2 μg/mL puromycin. To obtain BMMs, a single-cell suspension from the bone marrow from *Tet1^fl/fl^ Tet2^fl/fl^ Tet3^fl/fl^ Cre-ERT2* mice was cultured in the presence of 30 ng/mL macrophage colony-stimulating factor (M-CSF; PeproTech) for 3 days. To induce *Tet* gene deletion in cells derived from *Tet1^fl/fl^ Tet2^fl/fl^ Tet3^fl/fl^ ERT2-Cre* mice, the cells were treated with 4-hydroxytamoxifen (#H7904, 1 μM; Sigma-Aldrich) dissolved in ethanol for at least 2 days.

### 4.4. Cell Culture

The HEK 293 Tet-Off cells were purchased from Clontech and cultured in DMEM (12800-082; Gibco), supplemented with 10% FBS and 1 × penicillin/streptomycin. Leukemia cell lines including MOLM-14, HEL92.1.7, and U937 were cultured in RPMI (31800089; Gibco) supplemented with 10% FBS and 1 × penicillin/streptomycin. KG-1 was cultured in RPMI-1640 supplemented with 20% FBS and 1 × penicillin/streptomycin. All cell lines were subcultured every 2–3 days and maintained in a 10% CO_2_ incubator at 37 °C. For the experimental setups, MOLM-14 was plated at 5 × 10^5^ cells/mL, and HEL92.1.7, U937, and KG-1 were seeded at 2 × 10^5^ cells/mL.

### 4.5. Inducible Expression of TET1-CD and Generation of Monoclonal Cell Lines

The pTRE-Tight-FH-TET1-CD plasmid was constructed by inserting the cDNA encoding the Flag-HA (FH) tandem tagged with TET1-CD into a pTRE-Tight vector (Clontech). The HEK293 pTet-Off Advanced cell line (Clontech, Mountain View, CA, USA) was co-transfected with the pTRE-Tight-FH-TET1-CD and a Linear Puromycin Marker (Clontech). Subsequently, cells were subjected to selection using 1 μg/mL puromycin in the presence of 1 μg/mL doxycycline. To establish monoclonal cell lines, 200 cells from a stable cell pool were seeded onto a 100 mm dish and cultured over a period of 2–3 weeks. During this period, the culture media, containing 1 μg/mL puromycin and 1 μg/mL doxycycline, were replaced every 2–3 days. The isolation of individual colonies was achieved by gently rubbing each colony with 0.25% trypsin-soaked sterile filter paper and transferring them to separate wells of a 96-well plate. Upon sufficient expansion of each isolated cell clone, doxycycline was removed from the culture media, thereby inducing the expression of the transgene. This methodology ensured the creation of stable and homogenous monoclonal cell lines for subsequent experimental analyses.

### 4.6. Immunoblot Analysis

Cells were lysed with RIPA buffer (150 mM NaCl, 50 mM Tris–HCl, pH 8.0, 1% Triton X-100, 0.5% sodium deoxycholate, and 0.1% SDS), supplemented with a protease/phosphatase inhibitor cocktail (20 mM β-glycerophosphate, 10 mM sodium pyrophosphate, 1 mM sodium o-vanadate, 10 μM leupeptin, 10 μg/mL aprotinin, and 1 mM freshly prepared PMSF), and incubated on ice for 20 min. Cell debris was removed through centrifugation at 12,000 rpm for 15 min at 4 °C. Protein concentration was quantified by the Bradford protein assay. Subsequently, the cell lysates were mixed with SDS sample buffer and boiled for 5 min. Whole-cell lysates were separated by SDS-PAGE and transferred onto nitrocellulose membranes. Immunoblotting was performed in TBST (150 mM NaCl, 10 mM Tris–Cl, pH 8.0, and 0.5% Tween-20) supplemented with 5% low-fat milk and antibodies specific to the HA epitope (HA.11 clone 16B12; Covance, Princeton, NJ, USA) and actin (60008-1-Ig; Proteintech, Rosemont, IL, USA). Following primary antibody incubation, the membrane was probed with horseradish peroxidase (HRP)-conjugated secondary antibodies and visualized using enhanced chemiluminescence.

### 4.7. Immunocytochemistry

HEK293 Tet-Off cells were seeded at a density of 8 × 10^5^ cells/mL on sterile coverslips within 24-well plates. Following an overnight incubation, the cells were treated with the TET agonist candidates for 48 h. Subsequently, the cells were fixed with 4% paraformaldehyde in PBS for 15 min, followed by permeabilization with 0.2% Triton X-100 in PBS for an additional 15 min. Next, the cells were treated with 2N HCl at room temperature (RT) for 30 min to denature their DNA and neutralized using a 100 mM Tris–HCl solution (pH 8.5). After washing with PBS, the cells were blocked with 1% BSA and 0.05% Tween-20 in PBS at RT for 1 h. Then, primary antibody staining was performed by incubating the cells with a rabbit anti-5hmC polyclonal antibody (dilution 1:500) and a mouse anti-HA antibody (dilution 1:500) in the blocking buffer for 3 h at RT. Following three washes with permeabilization solution and an additional wash with PBS, the samples were incubated with donkey anti-rabbit Alexa 488 secondary antibody (dilution 1:1000) and donkey anti-mouse Alexa 555 (dilution 1:1000) in the blocking buffer at RT for 1 h. DNA was counterstained with 250 ng/mL of 4′,6-diamidino-2-phenylindole (DAPI) and mounted using SlowFade Gold Antifade Mountant (Molecular Probes, Eugene, OR, USA). The images were recorded using a Zeiss LSM 880 Confocal Microscope.

### 4.8. Dot Blot Analysis

Cells were treated with 200 μg/mL proteinase K (Roche) overnight at 55 °C and genomic DNA was isolated with equal volumes of phenol, phenol:chloroform:isoamyl alcohol (25:24:1), and chloroform:isoamyl alcohol (24:1), followed by precipitation with 2 volumes of ethanol. For the detection of cytosine-5-methylenesulfonate (CMS), genomic DNA was treated with sodium bisulfite using an EpiTect Bisulfite Kit (QIAGEN, Hilden, Germany). DNA samples were denatured in 0.4 M NaOH, 10 mM EDTA at 95 °C for 10 min, followed by neutralization with an equal volume of cold 2 M ammonium acetate (pH 7.0). Two-fold serial dilutions of the denatured DNA samples were spotted on a nitrocellulose membrane using a Bio-Dot apparatus (Bio-Rad, Hercules, CA, USA) following the manufacturer’s instructions. The membrane was subsequently washed with 2 × SSC buffer, air-dried, and vacuum-baked at 80 °C for 2 h, then blocked with 1 × Tris-buffered saline, 0.5% Tween-20, and 5% low-fat milk for 1 h. The membrane was incubated overnight with anti-5hmC or anti-CMS antibody (1:3000; provided by Dr. Anjana Rao, La Jolla Institute, La Jolla, CA, USA) at 4 °C. After incubating with HRP-conjugated anti-rabbit IgG secondary antibody, the membrane was visualized by enhanced chemiluminescence. To ensure equal spotting of total DNA on the membrane, the same blot was stained with 0.04% toluidine blue (89640-5G; Sigma-Aldrich).

### 4.9. In-Cell Western Analysis

In-cell Western was performed using standard immunocytochemistry protocol. HEK239T cells (4 × 10^4^) cells were plated in each well of an amine-coated BD PureCoatTM 384-well plate (BD Bioscience, San Antonio, TX, USA) and treated with drug compounds for 48 h. Cells were fixed with 4% paraformaldehyde solution for 15 min, washed with PBS, and permeabilized with 0.2% Triton X-100 in PBS. Then, DNAs were denatured with 2N HCl at RT for 30 min, neutralized with 100 mM Tris–HCl solution (pH 8.5), and incubated in a blocking buffer. After a 1 h incubation, a rabbit anti-5hmC polyclonal antibody (diluted to 1:5000; produced in-house) and a mouse anti-HA antibody (HA.11 clone 16B12, diluted to 1:4000; Covance) were added into the blocking buffer for 3 h at RT. After thorough washing with 0.2% Triton X-100 PBS, the cells were incubated at RT for 1 h with an IRDye 680RD-conjugated goat anti-rabbit IgG secondary antibody (926-68071, diluted to 1:4000; LI-COR Biosciences, Houston, TX, USA) and an IRDye 800CW-conjugated donkey anti-mouse IgG (926-32212, diluted to 1:6000; LI-COR Biosciences, Lincoln, NE, USA) in blocking buffer. The cells were then washed four times with wash buffer, followed by the addition of 50 μL of PBS. Fluorescence intensity was recorded using the Odyssey Infrared Imaging System (LI-COR). To quantify cell density, the cells were stained with IRDye 800CW maleimide antibody (929-80020, 4 ng/mL in PBS; LI-COR Biosciences) at RT for 15 min. After extensive washing with PBS three times, the plate was scanned once more and the staining was quantified using the Image Studio Software 5.5.4.

### 4.10. Lentiviral Production and Transduction

To produce lentivirus, HEK293T17 cells were transfected with lentiviral vectors and packaging constructs (pMD2.G and psPAX; Addgene, Watertown, MA, USA) using polyethylenimine (Polysciences, Warrington, Inc., Warrington, PA, USA). After 48 h, viral supernatant was collected and filtered through 0.45 µm low-protein binding filters. To establish a stable knockdown, cells were transduced with purified virus in the presence of 8 μg/mL polybrene, followed by selection with 10 µg/mL blasticidin (Invitrogen, Waltham, MA, USA). RNA interference (RNAi)-mediated knockdown of TET genes was achieved utilizing pLKO.1-Bla (Addgene) lentiviral vectors and the shRNA target sequences were the following: shTET1, 5′-CCTCCAGTCTTAATAAGGTTA-3′; shTET2, 5′-TTTCACGCCAAGTCGTTATTT-3′; and shTET3, 5′-GCGATTGCGTCGAACAAATAG-3′. The pLKO.1-Blast-Scramble was purchased from Addgene (#26701).

### 4.11. Quantitative Real-Time RT-PCR (qRT-PCR)

Total RNA was extracted utilizing TRIzol reagent (Life Technology, Carlsbad, CA, USA) following the manufacturer’s instructions. Equivalent amounts (0.5–1 μg) of total RNA were reverse-transcribed using SuperScriptTM IV Reverse Transcriptase (18090050; Invitrogen) in accordance with the manufacturer’s guidelines. Diluted cDNAs were analyzed by real-time PCR using QuantStudio 7 FLEX (Applied Biosystems, Waltham, MA, USA) using TOPrealTM qPCR 2 x PreMIX (SYBR Green with low ROX, RT500M; Enzynomics, Daejeon, Republic of Korea). The level of gene expression was normalized to *Gapdh*/*GAPDH*. The primer sequences are the following: *TET1* forward primer, 5′-TCTGTTGTTGTGCCTCTGGA-3′; *TET1* reverse primer, 5′-GCCTTTAAAACTTTGGGCTTC-3′; *TET2* forward primer, 5′-AAAGATGAAGGTCCTTTTTATA-3′; *TET2* reverse primer, 5′-TTTACCCTTCTGTCCAAACCTT-3′; *TET3* forward primer, 5′-CCATTGCAAAGTGGGTGA-3′; *TET3* reverse primer, 5′-CGCACCAGGCAGAGTAGC-3′, *GAPDH* forward primer, 5′-AGCCACATCGCTCAGACAC-3′; *GAPDH* reverse primer, 5′-GCCCAATACGACCAAATCC-3′; *Tet1* forward primer, 5′-GAGCCTGTTCCTCGATGTGG-3′; *Tet1* reverse primer, 5′-CAAACCCACCTGAGGCTGTT-3′; *Tet2* forward primer, 5′-AACCTGGCTACTGTCATTGCTCCA-3′; *Tet2* reverse primer, 5′-ATGTTCTGCTGGTCTCTGTGGGAA-3′; *Tet3* forward primer, 5′-CAACCCTCAGTGGCTTCTTG-3′; *Tet3* reverse primer, 5′-TGGGCCTTCATCTTTCTCCA-3′, *Gapdh* forward primer, 5′-GTGTTCCTACCCCCAATGTGT-3′; and *Gapdh* reverse primer, 5′-ATTGTCATACCAGGAAATGAGCTT-3′.

### 4.12. Cell Viability Assay

Cell viability was measured using a Quanti-Max™ WST-8 Cell Viability Assay Kit (QM5000; Biomax, Lublin, Poland) following the manufacturer’s instructions. This assay relies on the colorimetric quantification of orange-colored formazan, a product of dehydrogenase activity. Mitoxantrone was treated when cells were plated and after a 2-day incubation period, cell viability was assessed using the WST-8 assay according to the manufacturer’s instructions.

### 4.13. Statistical Analysis

The data are presented as the mean ± standard deviation (SD). Statistical significance was analyzed using a two-tailed, unpaired Student’s *t*-test using GraphPad Prism 8.0 software (GraphPad, San Diego, CA, USA). * *p* ≤ 0.05, ** *p* ≤ 0.01, *** *p* ≤ 0.001, and **** *p* ≤ 0.0001 were considered significant.

## 5. Conclusions

TET proteins stand out as potent epigenetic regulators, governing DNA demethylation processes and profoundly suppressing cancer initiation and progression. Our screening has proven invaluable in identifying novel TET activators, mitoxantrone and TBO, with identified candidates meriting further exploration as potential anti-cancer therapeutics. As loss of TET function is widespread in a wide range of cancers including hematological and solid cancers, we expect that the novel activators of TET proteins would probably find broad use in the treatment of cancers.

## Data Availability

All data generated or analyzed during this study are included in this article or can be requested from the author (M.K.) upon reasonable request.

## Supplementary Materials

The following supporting information can be downloaded at: https://www.mdpi.com/article/10.3390/ijms242216375/s1.
