# Supplementary material for: Development of Novel Epigenetic Anti-Cancer Therapy Targeting TET Proteins
Source: Int J Mol Sci. 2023 Nov 15;24(22):16375. doi: 10.3390/ijms242216375 (PMC10671484; doi:10.3390/ijms242216375)
Supplement: Supplementary file 1 [file ijms-24-16375-s001.zip › ijms-2696803-supplementary.pdf]

Supplementary information to:
Development of Novel Epigenetic Anti-Cancer Therapy Targeting TET Proteins

Hyejin Kim [1,†], Inkyung Jung [1,†], Chan Hyeong Lee [2], Jungeun An [2,*] and Myunggon Ko [1,3,*]

[1] Department of Biological Sciences, Ulsan National Institute of Science and Technology, Ulsan 44919, Republic of Korea; bpk0191@unist.ac.kr (H.K.); ikjung610@unist.ac.kr (I.J.)
[2] Department of Life Sciences, Jeonbuk National University, Jeonju 54896, Republic of Korea; chlee9243@jbnu.ac.kr
[3] Center for Genomic Integrity, Institute for Basic Science, Ulsan 44919, Republic of Korea
* Correspondence: jan@jbnu.ac.kr (J.A.); mgko@unist.ac.kr (M.K.); Tel.: +82-63-270-3356 (J.A.); +82-52-217-2516 (M.K.)
† These authors contributed equally to this work.

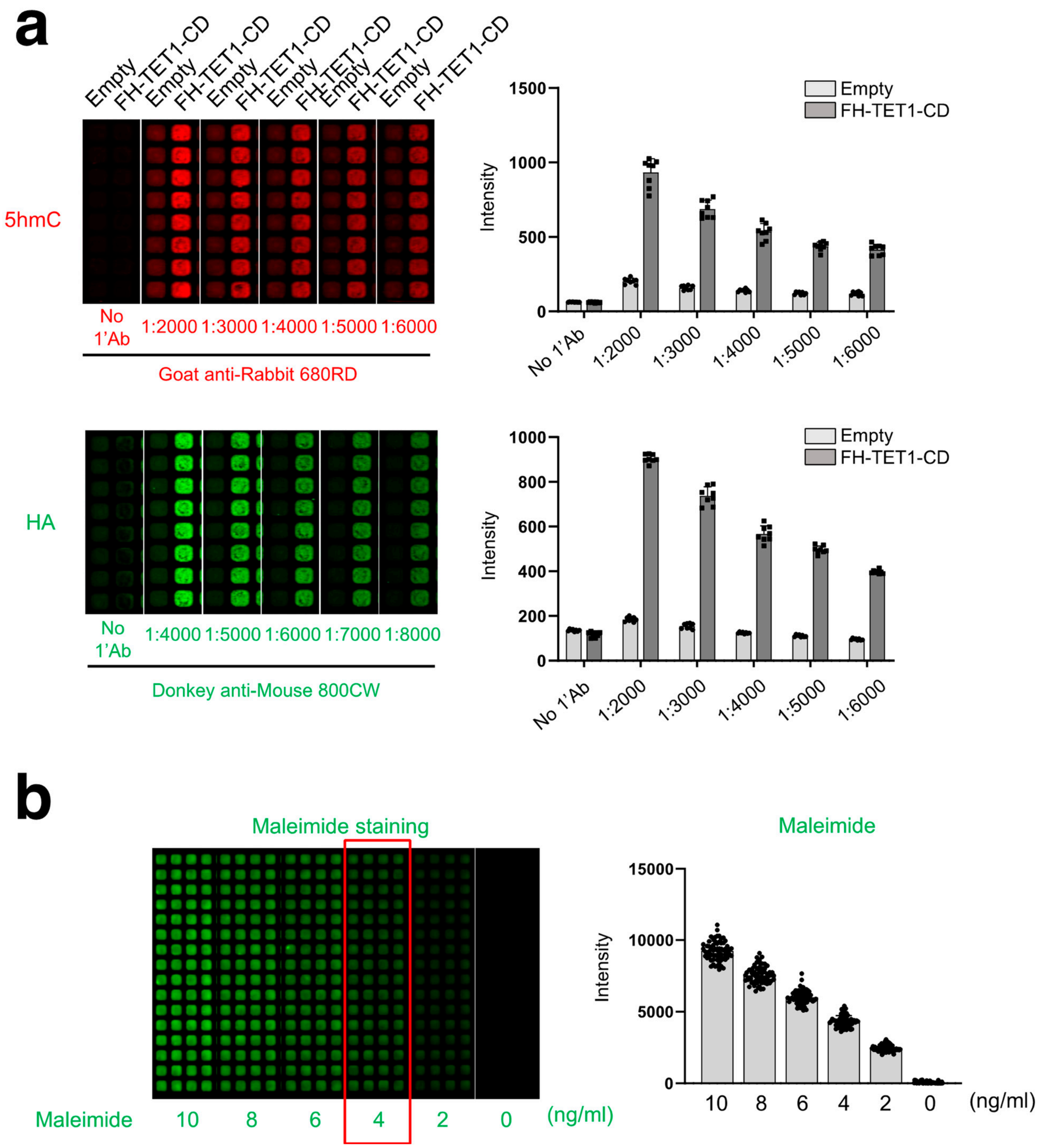

Figure S1. Optimization of the TET agonist screening system. (**a**) To optimize primary antibody titers, HEK293 cells transfected with empty vector or FH-TET1-CD were co-stained with varying concentrations of antibodies specific for 5hmC (red) and HA (green). A summary of intensities is shown on the right. (**b**) Determination of optimal concentration of maleimide. Cells were stained with sequentially diluted maleimide.

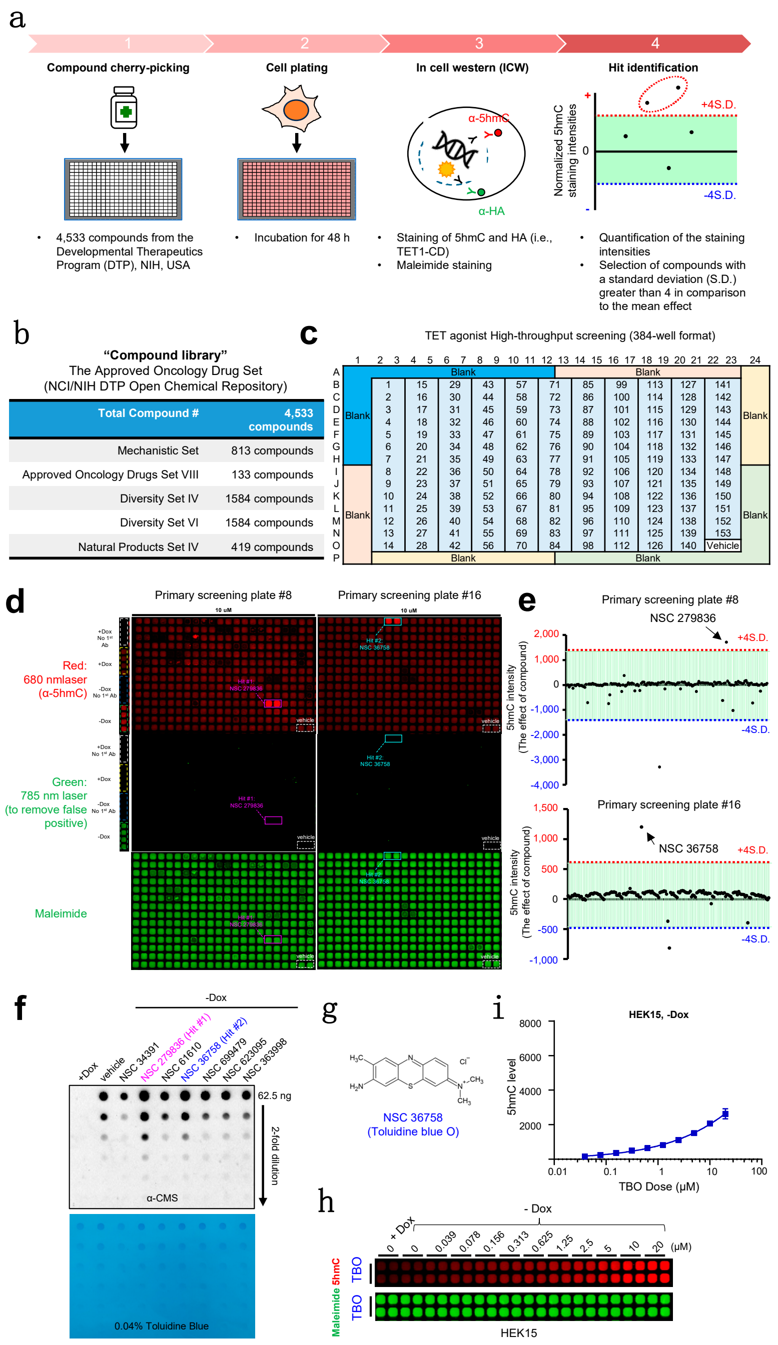

Figure S2. Identification of potential TET agonists through primary screening. (**a**) Flowchart of the screenin process. (**b**) Composition of the small molecule libraries used in this study. (**c**) A representative layout in a 384-well plate. (**d**) A representative example of staining results. Two identified hits were shown in the square. (**e**) Identification of NSC 279836 and NSC36758 as potential TET agonists based on the standard deviation (S.D.) greater than 4 in comparison to the mean effect induced by the compounds in each plate. (**f**) HEK#15 cells, cultured without Dox, were treated with indicated compounds for 48 hr, followed by 5hmC quantification using anti-CMS dot blot. Toluidine blue staining was used to monitor equivalent DNA loading. (**g**) Molecular structure of NSC36758, also known as Toluidine Blue O (TBO). (**h**) Dose-dependent increase in 5hmC intensities in HEK15 cells upon treatment with NSC36758. Maleimide staining was used to monitor compound-induced cytotoxicity. (**i**) Summary of the 5hmC signal intensities from (**h**). The 5hmC signal intensity was calculated by subtracting the mean of the +Dox group from that of drug-treated groups, which were then normalized to the mean of maleimide staining intensities.

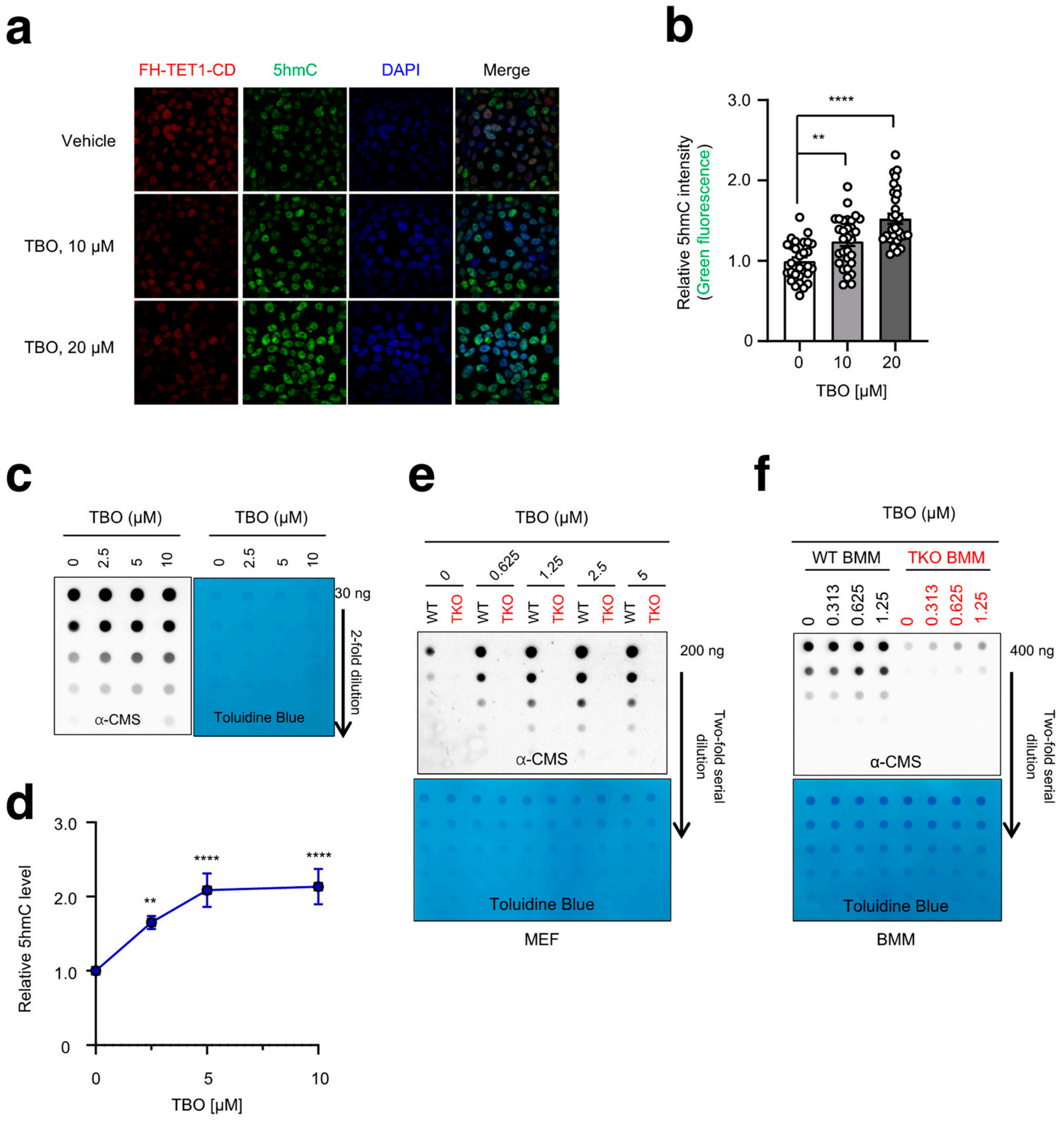

Figure S3. Toluidine Blue O (TBO) augments 5hmC levels in a TET-dependent manner. (**a**) Toluidine Blue O increases 5hmC by immunocytochemistry. HEK#15 cells, cultured without Dox, were treated with TBO at the indicated concentration and co-stained with antibodies specific for the HA epitope (red) and 5hmC (green). DAPI (blue) indicates nuclear staining. (**b**) Quantification of 5hmC intensities shown in (**a**). (**c**) Genomic DNA purified from HEK#15 cells treated with TBO as indicated was treated with bisulfite to convert 5hmC to CMS (cytosine 5-methylenesulfonate). CMS was quantified by dob blot assay using anti-CMS antibody. Toluidine blue staining was used to monitor equivalent DNA loading. (**d**) Summary of the 5hmC intensities shown in (**c**). (**e, f**) WT and TET TKO MEFs (**e**) or BMMs (**f**) were treated with increasing concentrations of TBO for 48 hr, followed by quantification of 5hmC by anti-CMS dot blot. Toluidine blue staining was used to monitor equivalent DNA loading. All data are presented as the mean ± s.d. The *P*-values were determined by unpaired Student's t-test. * **$P \leq 0.01$, ***$P \leq 0.001$, , and ****$P \leq 0.0001$.

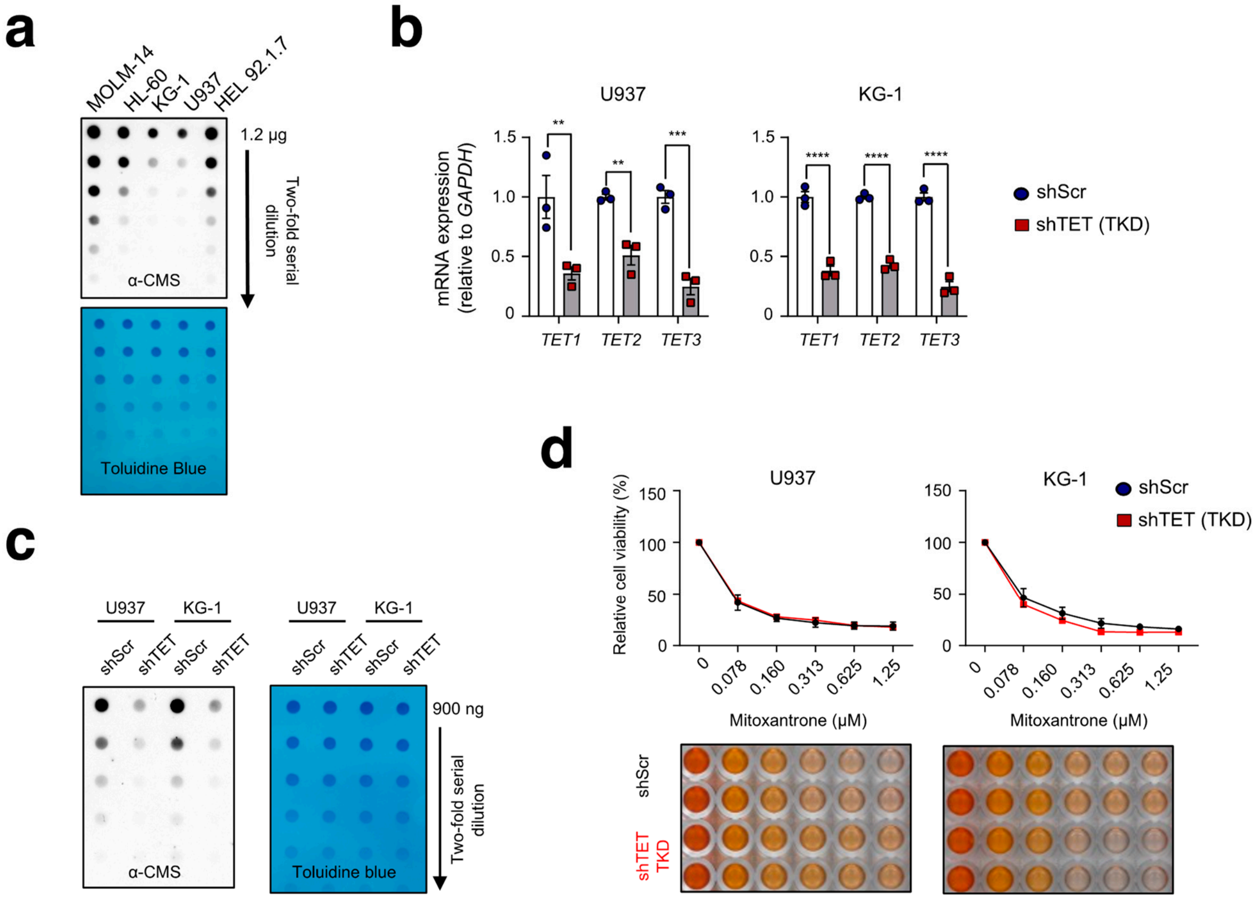

Figure S4. Mitoxantrone does not increase 5hmC levels in leukemic cell lines with low basal 5hmC levels. (**a**) Quantification of 5hmC levels in different leukemic cell lines by anti-CMS dot blot. Toluidine blue staining was used to monitor equivalent DNA loading. (**b**) Quantitative RT-PCR was performed to assess the levels of *TET1*, *TET2*, and *TET3* mRNAs relative to *GAPDH* in control and TET triple knockdown (TKD) U937 and KG-1 cells. n = 3. (**c**) Quantification of 5hmC by anti-CMS dot blot in cells shown in (**b**). Toluidine blue staining was used to monitor equivalent DNA loading. (**d**) No significant effect of mitoxantrone on cell viability of U937 and KG-1 cells. Control and TET TKD U937 (left) and KG-1 (right) cells were treated with mitoxantrone at the indicated concentrations. After 48 hr, cell viability was measured with WST-8 assay. All data are presented as the mean ± s.d. The *P*-values were determined by unpaired Student's t-test. * ***P* ≤ 0.01, ****P* ≤ 0.001, , and *****P* ≤ 0.0001.

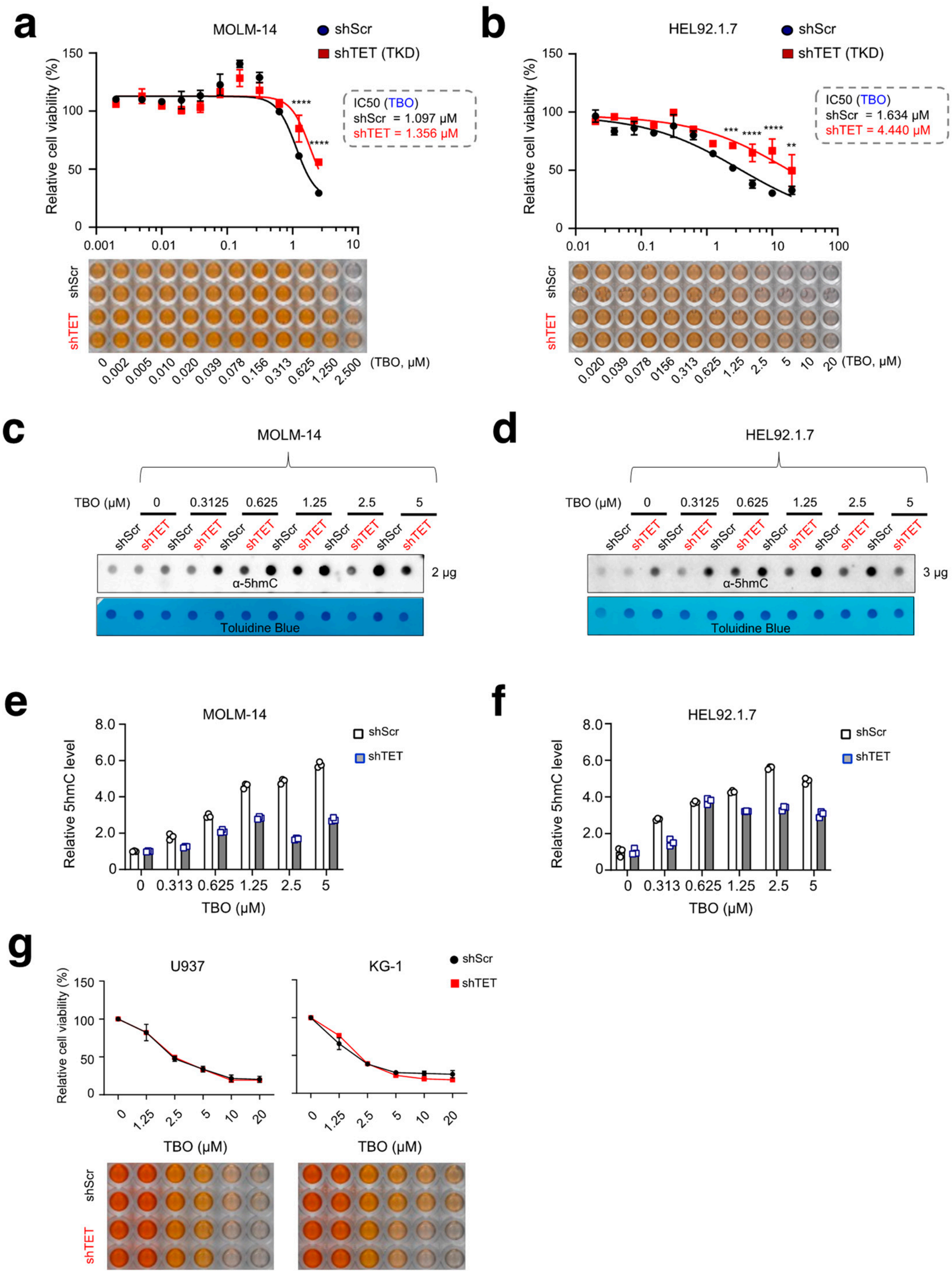

Figure S5. Toluidine Blue O (TBO) induces cancer cell death via TET activation. (**a**,**b**) The effect of TBO on cancer cell viability. Control and TET TKD MOLM-14 (**a**) and HEL92.1.7 (**b**) cells were treated with TBO at the indicated concentrations. After 48 hr, cell viability was measured with WST-8 assay. (**c**,**d**) Dose-dependent increase in 5hmC levels upon TBO treatment. Control and TET TKD MOLM-14 (**c**) and HEL92.1.7 (**d**) cells were treated with TBO at the indicated concentrations for 48 hr, followed by 5hmC quantification by anti-CMS dot blot. Toluidine blue staining was used to monitor equivalent DNA loading.(**e**,**f**) Summary of 5hmC levels shown in (**c**) and (**d**). (**g**) No significant effect of TBO on cell viability of U937 and KG-1 cells. Control and TET TKD U937 (left) and KG-1 (right) cells were treated with TBO at the indicated concentrations. After 48 hr, cell viability was measured with WST-8 assay.
